# Multicriteria Decision Analysis of the Influence of Natural Fibers on the Flexibility of Renewable Polyurethane Composites

**DOI:** 10.3390/ma18071610

**Published:** 2025-04-02

**Authors:** Edivane Cardoso, Viviane Escócio, Carlos Infante, Elen Pacheco

**Affiliations:** 1 Programa em Ciência e Tecnologia de Polímeros, Instituto de Macromoléculas, Universidade Federal do Rio de Janeiro, 2030 Horácio Macedo Av., Centro de Tecnologia, Block J, Rio de Janeiro 21941-598, Brazil; edivane.cardoso@ima.ufrj.br (E.C.); vivi75@ima.ufrj.br (V.E.); 2 Universidade Federal de São João del Rei, São João del Rei 36307-351, Brazil; prof.eduinfante@ufsj.edu.br; 3 Programa de Engenharia Ambiental, Escola Politécnica, Universidade Federal do Rio de Janeiro, 149 Athos da Silveira Ramos Av., Centro de Tecnologia, Block A, Ilha do Fundão, Rio de Janeiro 21941-611, Brazil

**Keywords:** natural fiber, polyurethane, renewable composite, flexible design, multicriteria decision analysis

## Abstract

Renewable polyurethane (PU) composites were developed using castor oil and long coir (LCF), ground coir (GCF) or cellulose fiber (CF) at PU/fiber ratios of 50/50, 60/40 and 70/30 wt/wt%, respectively. The aim was to study the influence of natural fibers on composite flexibility via thermogravimetry, differential scanning calorimetry, scanning electron microscopy and water absorption, density, tensile strength, flexural and flammability tests. The set of properties was evaluated (1) subjectively by assigning importance values to the different properties and (2) via multicriteria decision analysis (MCDA). In general, the PU composites with cellulose fiber (PU/CF) exhibited higher thermal degradation temperatures, greater tensile moduli and toughness and less flammability. The composites with the best results for both analysis methods (property set analysis) were PU/CF:60/40 wt/wt% and 70/30 wt/wt%, obtained with cellulose fiber (low lignin content) and the highest PU percentage; these were the most suitable for applications that require flexibility, such as in interior design. When comparing the different coir fiber sizes, the composites containing more long coir fiber (PU/LCF 60/40 wt/wt% and 70/30 wt/wt%) presented the best results. The results of subjective property set analysis were validated using multicriteria analysis, resulting in a simple analysis for application.

## 1. Introduction

Composites containing lignocellulosic fibers can be used for applications in different sectors (civil, automobile, aeronautical, textile, etc.). The main advantages of natural fibers in comparison to their synthetic counterparts are their low density and abrasiveness and their generally lower cost [1,2,3,4]. Additionally, using lignocellulosic waste makes composites more renewable [5,6,7]. Specifically, the design industry is searching for more renewable and flexible materials for use in rounded parts; that is, the material must not be damaged during bending. Designers also seek to combine color characteristics and mechanical, thermal and flammability properties [8].

The properties of composite materials depend on those of their components. Flexible matrices tend to produce flexible composites, such as polyurethane (PU) [9]; however, the filler used also affects flexibility [10]. In general, incorporating natural fibers reduces composite flexibility [1,11,12,13,14]. Thus, studies are needed to determine how lignocellulosic fillers can maintain PU flexibility based on the properties of composites.

Flexibility is the ability of a material to deform and return to its original shape when the applied force is removed. Flexible materials can be reversibly stretched when they undergo elastic deformation. The elastic limit of a material is characterized by its tensile strength, or the maximum amount of tensile stress it can withstand before breaking or permanently deforming [15].

The flexibility of composites is correlated with thermal and mechanical properties. With regard to thermal properties, the more flexible the composite, the lower its glass transition temperature (Tg). Composites with Tg values below ambient temperature are rubbery and flexible [16,17].

Regarding mechanical properties, flexibility can be correlated with the flexural modulus, flexural strength and toughness. The flexural modulus is related to the rigidity or stiffness of a material, where the higher the flexural modulus, the greater the stiffness and, consequently, the less flexible the composite, and the greater a material’s flexural strength, the lower its flexibility [14,15,16,18,19,20]. Toughness is related to a material’s ability to absorb energy until fracture. Thus, flexible composites should be tough to enable them to bend without fracturing [17].

Pinto et al. [14] studied the flexibility of PU- and cellulose-based composites with around 80 wt% of bacterial cellulose (from the genus *Acetobacter xylinum*) as filler. Despite also being a two-component material, the PU used to produce the composites was derived not only from castor oil but also from a mixture of polyols and other compounds, including propylene glycol, trimethylolpropane, butyl hydroxytoluene, xylene and ethyl glycol acetate. The composites showed flexibility, with excellent transparency and mechanical properties (tensile strength and tensile modulus up to 69 MPa and 6 GPa, respectively), with the potential for use in organic light-emitting diodes (OLEDs).

No other study was found that investigated the flexibility of castor oil-based PU composites containing lignocellulosic material. Other articles on castor oil-based PU composites describe properties such as flexural strength and are important for comparison with the results obtained here. Studies have investigated castor oil-based PU containing lignocellulosic fibers from plants [21] such as bamboo [11,22,23,24], curaua [25,26], banana [20,27], wood particles [28,29], coconut [30,31], sisal [32], sugarcane bagasse, sponge gourd [27], etc.

Composites consisting of bamboo and castor oil-based PU were produced by pressing, with PU ratios of 10, 15 and 20 wt%. The composites were submitted to physical (moisture content, swelling, water absorption and hardness) and thermal characterization [22]. Sánchez et al. [24] also studied the influence of the orientation and surface modifications of bamboo fibers on the physical and mechanical behavior of composites consisting of isocyanate and a polyester polyol obtained from castor oil, mixed at a ratio of 1:1.5. The focus area of the study was the civil construction sector. Bamboo fiber- and plant-based PU composites were developed to produce particleboard panels, and their density, absorption capacity, swelling rate and tensile, compressive and flexural strength were assessed [23]. Mothé et al. [25,26] produced composites using commercial PU and PU derived from castor oil and curaua fiber at proportions of 5, 10, 15 and 20 wt% of fiber. The tensile modulus was highest in the composite containing 80 wt% of commercial PU and 20 wt% of caraua fiber, reaching a maximum value of 98.3 MPa. The highest tensile strength (14.7 MPa) was recorded in the composite with 5 wt% fiber. Merlini et al. [20] studied the thermal and mechanical properties of a castor oil-based PU composite treated with sodium hydroxide solution and untreated banana fiber filler. The composites were produced by compression with fiber volume fractions of 5, 10 and 15 wt%/v. The composite with a treated fiber volume fraction of 15 wt% exhibited the highest tensile modulus, at 55.2 MPa, with a value of 5.9 MPa for pure PU.

Chen et al. [28] and Oliveira Jr. et al. [29] found that biobased PU composites can be used in the construction sector as a substitute for formaldehyde-based composites. These studies evaluated PU composites containing wood particles and wood flour, respectively.

Another important aspect is how to select the most appropriate composite for a specific application. Research generates many results based on the properties assessed, and selecting the most suitable composite for a given application is not a simple task. Additionally, a consolidated methodology is needed. Studies [33,34,35,36,37,38,39,40] employing multicriteria decision analysis (MCDA) are also used for decision making regarding composites. To this end, previously established criteria are used to select the ideal composition or process conditions, such as choosing the most appropriate natural fiber/polypropylene composite for automotive applications [33], assessing and selecting composite materials based on large-scale data [34] and selecting [36,38,39] and designing composite materials [35]. Life cycle assessment (LCA) can also be used in composite material selection [37].

Among the different MCDA methodologies [36], the preference ranking organization method for enrichment evaluation (PROMETHEE) is a recognized technique developed by Brans and Vincke in the early 1980s. According to Brans and Mareschal [41], PROMETHEE stands out for its ability to handle complex decision problems involving multiple criteria. The method allows decision makers to systematically evaluate and rank alternatives by considering both quantitative and qualitative factors.

Despite its widespread use and effectiveness, PROMETHEE has strengths and limitations. Its transparency and ability to incorporate stakeholder preferences are considered major advantages [42]. Additionally, guaranteeing the quality of input data and addressing model assumptions are essential in obtaining reliable results [43]. Further advantages are that it considers and compares all the alternatives; is simple, clear and stable; and does not involve normalization, which can minimize errors during decision making [36]. An important characteristic of PROMETHEE is its ability to assign weights (importance) to the criteria analyzed, enabling more robust and real pairwise comparison.

The aim of this study was to develop a flexible (foldable) renewable material for use in rounded parts such as furniture and domestic appliances or in the production of lamp frames. As such, the present study aimed to evaluate how natural fibers influence the flexibility of polyurethane (PU) composites synthesized with castor oil. To this end, several tests were conducted, and a subjective analysis was used for the first time, with a special focus on properties related to flexibility. This analysis was validated by MCDA to help select the composite with the best set of properties.

## 2. Materials and Methods

**Figure 1:**
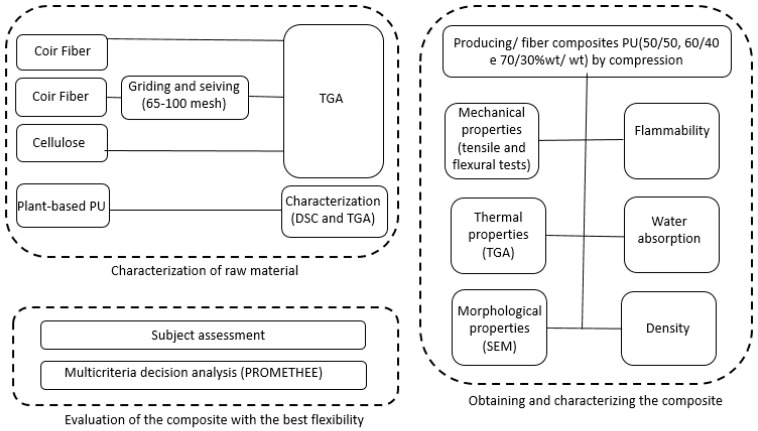
Flowchart of the main experimental stages.

Figure 1 shows a summarized schematic of the experimental procedure. The results of the experiment were analyzed (1) subjectively, with greater importance given to flexibility-related properties, and (2) via MCDA to validate the findings.

### 2.1. Materials

The raw materials used were as follows:

- IMPERVEG RM 122^®^ resin (PU) acquired from IMPERVEG, Aguaí, São Paulo state, Brazil. This two-component PU contains a polyol (component A) and a pre-polymer (component B). The polyol was synthesized from castor oil and the pre-polymer from methylene diphenylmethane diisocyanate (MDI), which was pre-polymerized with polyol, also derived from castor oil [44].
- Coir fiber from Bahia state was kindly donated by *Projeto Coco Verde* (in English: Green Coconut Project), Rio de Janeiro, Brazil. Two coir fiber sizes were used: long (LCF, long coir fiber) and ground (GCF, ground coir fiber).
- 99% pure commercial cellulose fiber (CF) (Fluff Pulp ECF (CAS No.: 9004-34-6)) was obtained from *Pinus taeda* trees and provided by Arauco Brazil.

**Figure 2:**
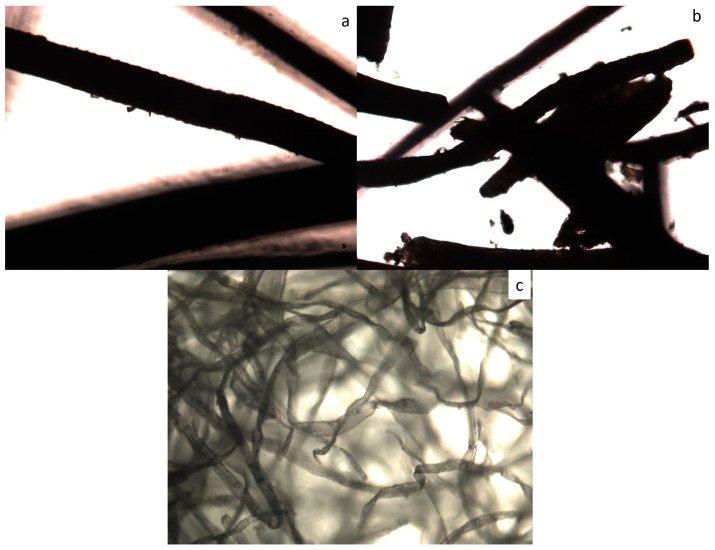
Optical microscopy images with 10× magnification: (**a**) long coir fiber; (**b**) ground coir fiber; (**c**) cellulose fiber.

The dimensions of the fibers are presented in Table 1. The width was measured using an optical microscope, and the length was measured using a pachymeter in ten specimens. Figure 2 shows optical microscopy images of the fibers. It can be seen that coconut fibers are more irregular than cellulose fibers.

### 2.2. Chemical Composition of the Natural Fibers

#### 2.2.1. Holocellulose Content

Holocellulose content was determined in duplicate according to the TAPPI T19m-54 standard, adapted for lignocellulosic fibers [45]. This experiment was conducted for the ground coir (65–100 mesh) and cellulose fiber samples. A 3.00 g sample was dried in an oven at 60 °C for 24 h, and then 150 mL of distilled water, 1.0 mL of glacial acetic acid and 2.5 g of sodium chlorite were added, and the sample was maintained in a bath at 70 ± 2 °C under constant agitation for an hour. This procedure was repeated an additional two times. The mixture was cooled in ice after 3 h and then filtered and dried. The filtrate was thoroughly washed with distilled water until pH 7. Then, it was placed in an oven at 60 °C for 24 h and weighed until constant mass. In this process, lignin is eliminated, and the final sample is composed of holocellulose (cellulose and hemicelluloses) [45].

#### 2.2.2. Hemicellulose and α-Cellulose Content

Hemicellulose was calculated based on the difference between holocellulose and α-cellulose content [45]. First, 1 g of holocellulose was weighed (procedure described in Section 2.2.1) and placed in 10 mL of 17.5 wt/wt/% sodium hydroxide solution for 2 min. Then, it was ground for 8 min and allowed to rest for 20 min. The material was filtered and washed with distilled water until pH 7.0. Subsequently, the filtrate was washed with 200 mL of 20 wt%/v of acetic acid followed by 200 mL of distilled water. The material (α-cellulose) was placed in an oven at 60 °C for 24 h and then weighed to obtain the α-cellulose dry weight.

#### 2.2.3. Acid-Insoluble Lignin Content

The acid-insoluble lignin content was determined in duplicate for each fiber type (GCF and CF) via the Klason method (TAPPI T13M-54), which is based on the hydrolysis of polysaccharide acids, and gravimetric analysis by separating acid-insoluble lignin after a reaction in sulfuric acid (72 *v*/*v*%). First, a 1.0 g sample was weighed, ground in a pestle with 15.0 mL of 72 *v*/*v*% sulfuric acid solution, and then allowed to rest for 24 h. Then, the material was transferred to a 1.0 L flask, and 560 mL of distilled water was added, followed by reflux for 4 h, thus yielding the acid-insoluble lignin present in the sample. The lignin was filtered and then washed abundantly with distilled water. Finally, the lignin was dried in an oven at 105 °C until constant mass. Lignin content was calculated according to [27,45].

### 2.3. PU Curing Assessment

The curing temperature of the composites was established by differential scanning calorimetry (DSC) via kinetic analysis of the curing reaction using a Hitachi Thermal Analysis System (DSC 7020, TA7000) under a nitrogen (N_2_) atmosphere at a heating rate of 10 °C·min^−1^ from 20 to 150 °C for a sample weight of 6.000 mg before curing. The polyol (component A) and pre-polymer (component B) were mixed in the sample container at the moment of analysis. The maximum curing temperature was determined as the peak of the exothermic curve [46].

### 2.4. Obtaining the Composites

The PU was obtained before being mixed with the natural fiber. Components A (polyol) and B (pre-polymer) were mixed at an A/B ratio of 2:1 wt/wt%, in accordance with the manufacturer’s instructions [44].

The composite plate was produced by compression in a hydraulic press with mold dimensions of 10 cm × 10 cm × 3 mm. Before pressing, the natural fibers were dried in an oven at 60 °C for 24 h. The PU/fiber ratios used were 50/50, 60/40 and 70/30 wt/wt%.

The methodology used to mold the composites was based on Temer and D’Almeida [12]. Since CF and LCF are bulky, these fibers were prepressed at ambient temperature under a pressure of 5 t for 10 min before adding PU.

Initial prepressing was not performed for the GCF composites. The weighed ground fiber was manually mixed with PU in a container before pouring the mixture into the mold. The same molding conditions were applied for all composites. Then, the PU was spread evenly over the prepressed fiber.

Curing temperature and pressure were determined experimentally. The curing temperature was chosen based on the results of DSC analysis of curing enthalpy (Section 2.3). After curing, the plate was removed from the press and cooled at ambient temperature. Table 2 shows the amount by weight of raw material (components A and B and lignocellulosic fibers) used to prepare the composites.

### 2.5. Thermogravimetric Analysis

Thermogravimetric analyses (TGAs) of the natural fibers, PU and composites were performed at a heating rate of 10 °C·min^−1^ from 25 to 800 °C under a N_2_ atmosphere with a flow rate of 40 mL·min^−1^ in a thermogravimetric analyzer (TA Instruments, New Castle, DE, USA, Q500). The weight of each sample was approximately 10 mg.

### 2.6. Mechanical Properties

Mechanical properties were analyzed using an EMIC DL 3000 universal testing machine.

#### 2.6.1. Tensile Test

Type 4 test specimens were cut in accordance with ASTM D638-10 and tested at a grip separation speed of 3 mm·min^−1^, grip separation of 25.4 mm and 5 kN load cell. The result was determined as the median of 5 test specimens. Toughness was calculated from the area under the stress-versus-strain curve [47].

#### 2.6.2. Flexural Test

This test was carried out in line with ASTM D790-10 for materials 3.0 mm thick, 45 mm long and 12.7 mm wide. The grip separation was 32 mm, and the testing speed was 0.85 mm·min^−1^.

### 2.7. Morphological Properties

The composites and PU were morphologically analyzed by scanning electron microscopy (SEM) using a Joel JSM-6510LV scanning electron microscope. The samples were prepared by cryogenic fracture followed by gold metallization. Electron acceleration was 20 kV.

### 2.8. Density

The density of the composites and pure PU was determined out in accordance with ASTM D 792-13. The test specimens (2 cm × 2 cm × 3 mm) were weighed in ethyl alcohol with a density of 0.789 g·cm^−3^ (20 °C). The test was performed in duplicate, and the result was the average of the analyses.

Coir fiber density is between 1.1 and 1.5 g/cm^3^ [48] and, according to Marinelli et al. [49], that of cotton fiber is between 1.5 and 1.6 g/cm^3^. The estimated theoretical density was calculated based on the average values reported in the literature [48] for coir fiber (1.3 g/cm^3^) and cellulose fiber (1.5 g/cm^3^) [50]. Theoretical density values were calculated based on these results for comparison with density, explained experimentally via Equation (1).

(1) $$Theoretical\;density=\left(PU\;density\times PU\;\%wt\right)+\left(Fiber\;density\times Fiber\;\%wt\right)$$

### 2.9. Water Absorption

The water absorption was analyzed according to ASTM D570-18. The test specimens were cut to a size of 3.0 mm × 2.0 cm × 2.0 cm × 3 mm. They were submerged in a beaker with 500 mL of boiling distilled water for 2 h and then transferred to another beaker containing 500 mL of distilled water at ambient temperature. Finally, the samples were removed from the water and patted dry with paper towel. The samples were weighed on an analytical balance before and after submersion. The tests were performed in duplicate.

### 2.10. Flammability

The composites and pure PU were analyzed according to UL 94 flammability tests (method A), placed horizontally in a flammability tester (Fire Testing Technology) at a 45° burner angle. The samples were 10 cm long, 1.3 cm wide and 3.0 mm thick. Five tests were conducted for each composite composition.

Burning time and the burning characteristics of each component of the composite were monitored. Burning time was measured after the flame from the Bunsen burner had been removed. Equation (2) was used to calculate the burn rate of the test specimens.

(2) $$V=\frac{60L}{t}$$

where V = burn rate (mm·s^−1^), L = burned length (mm) and *t* = burning time (s).

The burning time used in Equation (2) was the average value, obtained as the arithmetic mean of the burning times for 5 test specimens. For the average burn rate (mm·s^−1^), the average burning time and burned lengths (mm) were used, with the latter calculated based on the arithmetic mean of the burned lengths of 5 tests specimens.

### 2.11. Analysis of Composite Properties with an Emphasis on Flexibility

The analysis of the set of properties was carried out in two ways, including one simple method (subjective analysis) and another that requires software (multicriteria decision analysis—PROMETHEE).

#### 2.11.1. Subjective Analysis

In the present study, a subjective analysis of the set of properties for the best-performing composites was performed, with special emphasis on flexibility-related properties. This analysis is used in environmental impact assessment [50,51,52].

Composites for application in civil construction, specifically in interior design, should exhibit flexibility-related properties such as flexural strength, flexural modulus and toughness, specifically a low flexural modulus and flexural strength [14,15,18,19,20]. However, values for these properties cannot be very low since fracture is undesirable. It is also important to consider the results of TGA, SEM, tensile modulus, density, water absorption and flammability. This set of properties was tabulated for better visualization and calculation, with greater importance given to those linked to flexibility.

In TGA, thermal stability was considered from the initial degradation temperature (Tonset) of the composites [17,20,26]. SEM provided a comparative analysis of the presence of bubbles in the composites. This result was not expressed numerically but rather based on a visual comparison of the micrographs. For mechanical properties (flexural and tensile tests), the median values were tabulated, since these were used for calculations, while for the remaining properties (density, water absorption and flammability), the tabulated values correspond to the mean.

Once tabulated, the mean value for each property was calculated, considering the results of all the compositions, in order to identify values that were more or less positive. The values for the properties were classified as follows:

✓ More positive—those most beneficial to the property, shown in green for better visualization.
✓ Less positive—those least beneficial to the property, shown in red.
✓ Median—values between the most or least positive (mean values), shown in blue.

Once values had been classified as more or less positive or median, subjective importance values from 1 to 3 were attributed to the properties. Importance values related to flexibility were the highest, since this is the most desirable property. The classification was as follows:

✓ Properties with an importance value of 3: Related to flexibility (toughness, flexural strength and flexural modulus), with values also classified as more (+) or less positive (−). Comparatively, more flexible materials exhibit lower flexural strength and flexural moduli [14,15,16,18,19,20].
✓ Properties with an importance value of 2: Related to flexibility (toughness, flexural strength and flexural modulus), classified as median or mean, considered (+).
✓ Other properties studied with an importance value of 2: Tonset in TGA, tensile modulus, presence of bubbles in SEM, density, water absorption, burning time and burn rate in flammability tests, the values of which were more (+) or less positive (−).
✓ Other properties with an importance value of 1: Tonset in TGA, tensile modulus, presence of bubbles in SEM, density, water absorption, burning time and burn rate in flammability tests, the values of which were classified as median or mean, considered (+).

Finally, an additional table was compiled showing the importance values (±1, ±2 or ±3) of each composite, which were summed. Composites with the highest positive values were deemed to have a set of properties best suited to applications requiring greater flexibility.

#### 2.11.2. Multicriteria Decision Analysis

The main function of PROMETHEE is to model the preferences of the decision maker, which are represented by functions that indicate the degree of preference for one alternative over another in relation to a specific criterion. Weights reflect the relative importance of each criterion in the global assessment of the alternatives, and pairwise comparison is a key process in PROMETHEE. The pairs of alternatives are compared for each criterion using the corresponding preference function [53].

The PROMETHEE stages were as follows:

- Assessment framework:

A decision framework was compiled in line with the PROMETHEE method, explaining the values of each composite for each specific property.

- Preference functions:

The analysis pattern used for the PROMETHEE preference functions was in accordance with the type of data. In the present study, the usual function (type I) was adopted because the data were predominantly quantitative [53].

✓ Description: The simplest function, whereby any positive difference between the alternatives is considered a global preference.
✓ Application: Used when the difference between assessments of the alternatives is significant.
✓ Formula: P (d) = 0 if d ≤ 0; P (d) = 1 if d > 0.

Where P (d): Preference index, from 0 to 1; d: difference between assessments of two alternatives in relation to a specific criterion.

- Weights of the properties:

Weights from 1 to 4 were assigned to the properties analyzed based on their relative importance to the study, with 1 indicating the smallest impact and 4 the highest. Table 3 shows the weights attributed to each property.

Correctly determining the weights allows decision makers to balance the trade-offs between them and more accurately establish their preferences and priorities. As such, analyzing weights is essential to ensure that the final solution is robust, consistent and in line with stakeholders’ interests. Importance is not related to the range of the ranking scale but rather to the impact of the weight attributed. The ranking attributed must correspond to the robustness index in order to minimize uncertainty in the result [42]. The scale from 1 to 4 accurately reflects this minimization of risk in the results, contributing to an analysis of importance capable of satisfying the relationships between the properties.

The images generated in the MCDA results were obtained using Visual PROMETHEE software, which is freely accessible. Visual PROMETHEE is a widely used tool for implementing the PROMETHEE method, allowing for a comprehensive analysis and visualization of decision-making processes.

## 3. Results

### 3.1. Assessment of Curing Time by Differential Scanning Calorimetry

**Figure 3:**
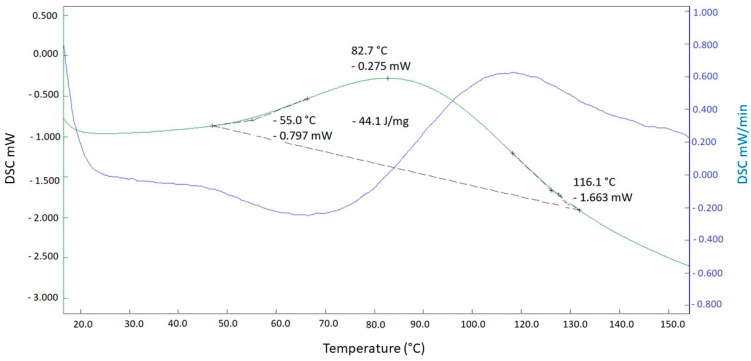
Differential scanning calorimetry curve (green) and its derivative (blue) for PU curing temperature.

Differential scanning calorimetry (DSC) was used to assess the curing time of the PU used in composite molding. The results of the first heating curve were used because by the second curve, the PU had already been cured. Figure 3 shows the first heating curve and its derivative.

The temperature at the start of the curve (onset of PU curing) was 55 °C, peaking at 82.7 °C and ending at 116.1 °C. In other words, PU curing began at 55 °C and peaked at 82.7 °C. As such, the temperatures tested to assess curing were 55, 60, 65, 70, 75, 80 and 85 °C.

The composites were molded at 60 °C, since this was the lowest temperature that allowed for the proper demolding of the pressed composite sheet. Demolding was performed after cooling for 24 h at ambient temperature.

### 3.2. Chemical Characterization of the Lignocellulosic Fibers

Table 4 shows the results for holocellulose, α-cellulose, hemicellulose and acid-insoluble lignin content for coir and cellulose fibers.

Cellulose fiber exhibited a higher holocellulose content than coir, while the latter displayed the highest lignin content. The results obtained for coir fiber corroborate those reported in the literature [3,4,31,54].

Lignocellulosic coir fiber is obtained from the fibrous mesocarp of the fruit of the coconut palm (*Cocos nucifera*), widely distributed in tropical zones. Its main characteristics are its low density, high availability, dark color and considerable durability due to its relatively high lignin content when compared with other natural fibers [31,55,56]. Coir fiber contains around 27–44% cellulose, 40–49% lignin and 0.2–14% hemicellulose [3,4,31].

As mentioned in the description of the material in the methodology section, cellulose is extracted from *Pinus taeda* trees [57]. Even after extraction, 2.0 ± 1.4% of lignin remained in the cellulose fiber.

### 3.3. Obtaining the Composites

Table 5 shows photographs of the pressed plate of PU/fiber composites: 50/50, 60/40 and 70/30 wt/wt%. The coir fiber composites are darker than those containing cellulose fibers. The high lignin content of coir fibers gives these composites a darker color, similar to that of the wood-fiber composite [58]. However, ground coir fiber has a larger surface area, resulting in greater exposure of the lignin, which likely makes these composites even darker than those containing long coir fiber.

### 3.4. Composite Characterization

#### 3.4.1. Thermal Properties of the Composites

Table 6 shows the initial degradation temperature (Tonset) and percentage of undegraded waste at 800 °C for the raw materials and PU/fiber composites. Theoretically, GCF and LCF should have similar degradation temperatures; however, differences were observed between Tonset values, with peaks at 15 and 9.2 °C, respectively. Comparatively, GCF has a larger surface area and therefore greater access to heat for thermal degradation. As such, these fibers likely degrade at lower temperatures than LCF. Similar initial mass loss values were recorded for GCF and LCF at 100 °C of 7.4% and 8.7%, corresponding to low-molecular-weight molecules such as moisture and waxes present in the fiber composition [59,60,61].

Among the plant fibers used, GCF showed the highest thermal stability, followed by LCF, with cellulose fiber as the most thermally stable. The castor oil-based PU resin displayed better thermal stability than the LCF, and the PU resin was less thermally stable than the cellulose fiber.

In general, the Tonset results were similar for composites containing the same fiber, ranging from 289 and 326 °C. In relation to the type of fiber, the cellulose fiber composites exhibited the highest thermal stability. The coir fiber composites displayed similar Tonset values (288–299 °C) to those of PU (Tonset = 294 °C), whereas higher temperatures were recorded for cellulose fiber composites (318–326 °C).

#### 3.4.2. Mechanical Properties

(a) Tensile tests

Ultimate Tensile Strength

**Figure 4:**
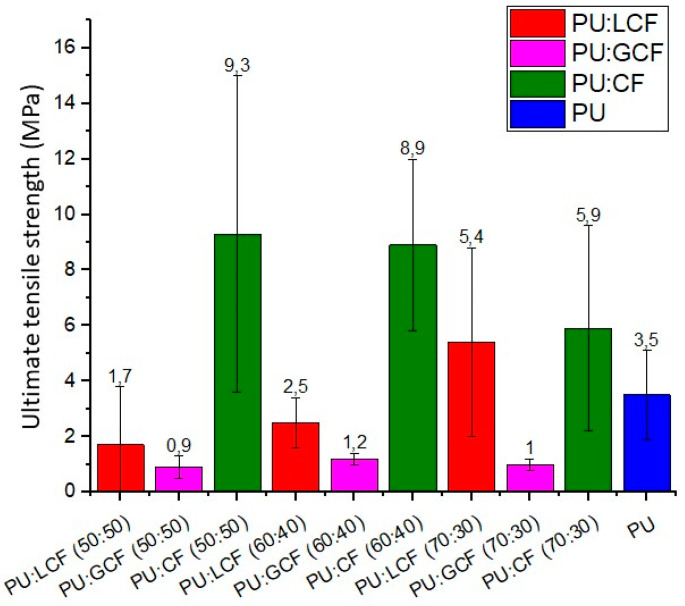
Ultimate tensile strength of PU and PU/LCF, PU/GCF and PU/CF composites.

Figure 4 shows the ultimate tensile strength results for PU composites containing coir and cellulose fiber. The highest tensile strength values were recorded for the CF composites and the lowest for their GCF counterparts. Ultimate tensile strength was higher in LCF than in GCF composites.

The effects of fiber composition variations on ultimate tensile strength differed between composites. A larger amount of fiber resulted in higher ultimate tensile strength values for CF composites but lower values for LCF composites. Ultimate tensile strength did not differ with increasing fiber content in the PU/GCF composite.

The performance of CF composites and those containing 30 wt% LCF was superior to that of PU. Longer fibers absorb more energy, resulting in better properties of the composite [4]. Another noteworthy point is the higher modulus of CF, which responds more positively to mechanical properties than fibers with a higher lignin content, such as coir fiber [3]. The reported tensile modulus for cotton fiber is between 3 and 12.6 GPa [1,54], while that of coir fiber is lower, at 3.0 to 7.0 GPa for LCF [1,31,54].

Faria et al. [30] developed PU and LCF composites with fiber concentrations of 30, 40%, 50% and 60% by volume and reported tensile strength values between 6.5 and 6.7 MPa, which did not vary with an increase in fiber content. In the present study, this stability was observed in the GCF composite.

It is important to note that some results exhibited high standard deviations, at times higher than the value obtained. This is due to the bulkiness and unevenness of the natural fibers (Table 1), making it difficult to produce homogeneous pressed plates with more homogeneous property results.

Toughness

Table 7 shows the toughness values obtained. Fiber incorporation reduced the toughness of the PU. The toughest composites were those that contained CF.

The LCF composites were tougher than their GCF counterparts at all the PU/fiber ratios studied. This can be justified by the greater stress resistance of long fibers compared to that of particulate fibers [3,4]. While toughness was higher at high fiber contents in CF composites, the toughest LCF composites were those with the highest fiber content. The PU/CF 50/50 wt/wt% composite exhibited the highest toughness. As observed for ultimate tensile strength and maximum deformation, there was no linearity between a higher or lower fiber content and higher or lower toughness in the GCF composites.

Tensile Modulus

Table 7 presents the tensile moduli of the composites. No tensile modulus values were detected by the universal testing machine for the 50/50 wt/wt% PU/GCF composites under the analysis conditions. These composites were the most fragile and brittle of all those tested, likely due to the high fiber content of the PU matrix, resulting in poor strength and hampering tensile modulus detection.

Tensile moduli were higher in LCF than in GCF composites, thus confirming that the LCF composites and all the PU/fiber ratios were stiffer than their GCF counterparts. Adding CF to PU significantly increased the tensile modulus, with the composites containing CF obtaining the highest values. As previously mentioned, the tensile modulus reported for cotton fiber is greater than that of coir fiber [1,31,54]. Thus, cellulose fiber was expected to provide a higher tensile modulus in composites than coir fiber.

In LCF composites, the tensile modulus increased as fiber content declined, with the 50 and 30 wt/wt% composites exhibiting the highest and lowest values, respectively.

Hadjadj et al. [62] reported a significant increase in the tensile moduli of composites containing cellulose (extracted from Alfa stems) and PU synthesized from e-caprolactone, 1,6-hexamethylenediisocyanate and bis-hydroxy ethylene terephthalate. The 250–700% increase observed with 5% and 30% fiber, respectively, was attributed to the high-modulus fiber incorporated into the PU. Likewise, the tensile modulus results presented in Table 7 are higher than those obtained for pure PU.

Pinto et al. [14] studied the tensile modulus of PU composites prepared from castor oil and bacterial cellulose and recorded a modulus of 6 × 10^3^ MPa for the 79% cellulose composition and 16 MPa for pure PU.

(b) Flexural tests

Flexural strength

Table 8 shows the flexural strength values of the composites. Flexural tests could not be conducted for the pure PU because the plates were too flexible.

The CF composites exhibited the highest flexural strength values, whereas the GCF composites exhibited lower values. Flexural strength tended to decline as fiber content decreased in LCF and CF composites. However, this linear trend was not observed for the GCF composites, with the lowest strength exhibited by the composite with a 60/40 wt/wt% PU/fiber ratio. According to the literature [63], with an increase in the coconut content in PU composites, there is a decrease in flexural strength, from 6 × 10^−2^ MPa with 5% fiber to 9 × 10^−3^ MPa with 20% fiber. In addition, the values presented in the literature are lower than those found in this work.

Flexural Modulus

On comparison of the fiber types, the cellulose fibers exhibited the highest flexural modulus at all the ratios tested, and the GCF composites exhibited the lowest (Table 8). The 50/50 and 60/40 wt/wt% PU/CF composites exhibited the highest flexural modulus values. Composites with long coconut fibers showed higher elastic modulus values than composites with ground coconut fiber in all proportions (Table 8). Fiber size also plays an important role in the mechanical properties of composites [63]. For CF and LCF composites, the lower the fiber content, the lower the flexural modulus; however, this relationship was not observed for GCF in flexural or tensile testing.

Liu et al. [21] analyzed composites prepared with thermosets, including acrylated epoxidized soybean oil (AESO) and AESO with methacrylated isosorbide (MI) as a comonomer (MI-AESO), using bamboo and hemp fibers as reinforcement at a ratio of 1:1. The bamboo fiber composites were more flexible than those reinforced with hemp fibers. The bamboo and hemp fiber-reinforced AESO resin composites obtained elastic moduli of approximately 2.5 × 10^3^ MPa and 5.3 × 10^3^ MPa, respectively, and 5.8 × 10^3^ MPa and 10.3 × 10^3^ MPa for the MI-AESO composites also reinforced with bamboo and hemp fibers.

#### 3.4.3. Morphological Analysis of the Composites

**Figure 5:**
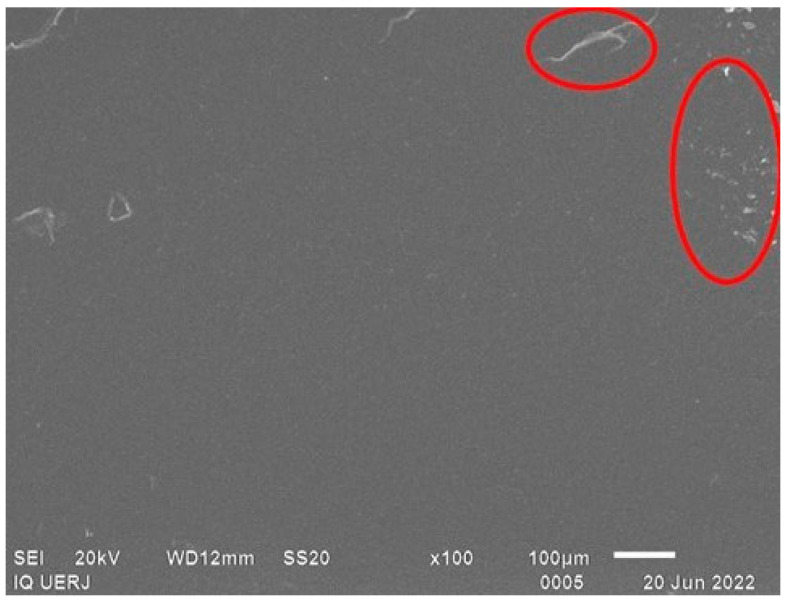
Micrograph of polyurethane. Note: Cracks circled in red.

**Figure 6:**
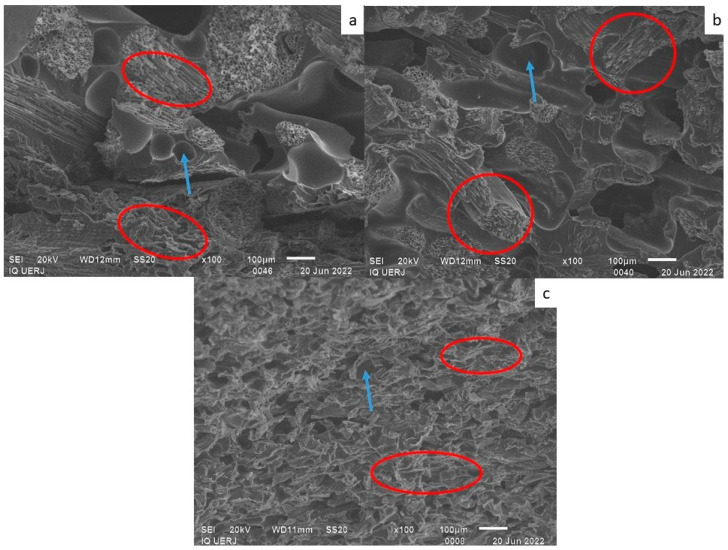
Micrographs of the 50/50 wt/wt% PU/fiber composites containing (**a**) long coir fiber; (**b**) ground coir fiber; (**c**) cellulose fiber. Note: Red circle: fibers; blue arrow: bubbles.

**Figure 7:**
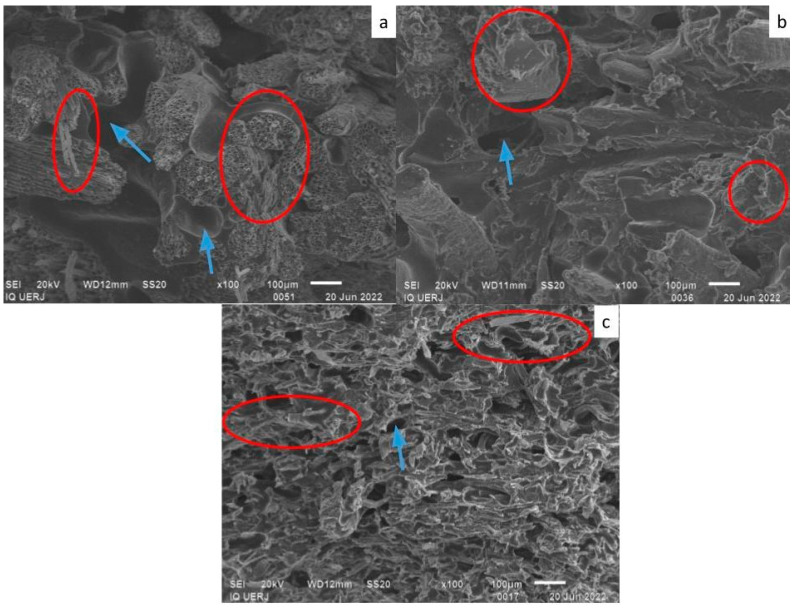
Micrographs of the 60/40 wt/wt% PU/fiber composites containing (**a**) long coir fiber; (**b**) ground coir fiber; (**c**) cellulose fiber. Red circle: fibers; blue arrow: bubbles.

**Figure 8:**
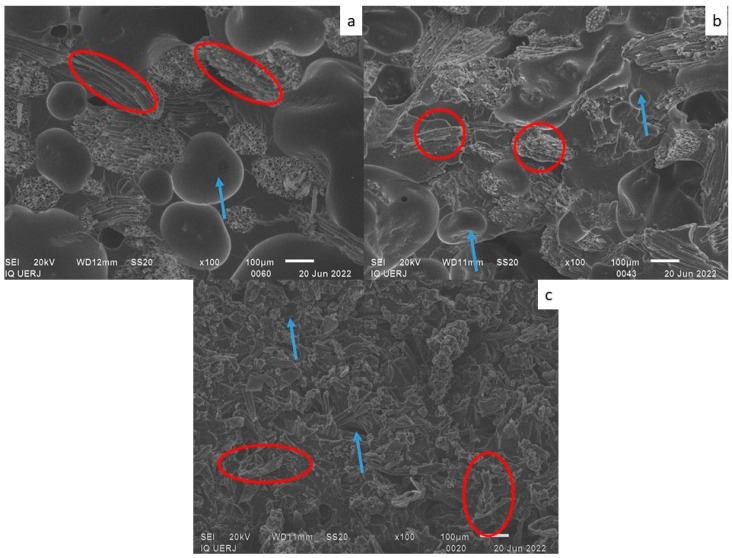
Micrographs of the 70/30 wt/wt% PU/fiber composites containing (**a**) long coir fiber; (**b**) ground coir fiber; (**c**) cellulose fiber. Red circle: fibers; blue arrow: bubbles.

Figure 5 shows an SEM micrograph of PU at ×100 magnification. Small cracks and bubbles can be observed in the PU (circled in red). These cracks may be due to cryogenic fracture for sample preparation, which occurred before SEM analysis. Figure 6, Figure 7 and Figure 8 shows micrographs of the 50/50, 60/40 and 70/30 wt/wt% PU/fiber composites, respectively, all at ×100 magnification. In the images, some fiber fragments are circled in red, and bubbles are indicated by a blue arrow.

In general, the coir fiber composites contained larger bubbles, while those in the CF composites were visually smaller. Another noteworthy point is that the fibers in general seem to be adhered to the PU matrix, that is, there are no spaces between the fibers and matrix.

The bubbles clearly visible in the composites were less obvious in the pure PU. In the composites, these bubbles may originate from the hydrophilic natural fibers, despite being dried. Another possibility is that bubbles form due to the PU curing reaction that produces CO_2_ as a byproduct [9]; however, this seems least probable, since there were no bubbles in the pure PU.

Despite the smaller bubbles in the cellulose fiber composites, the fibers appear well distributed in the matrix, which may also justify some of the favorable results regarding mechanical properties.

Composites with coir fiber, both long and ground, seemed to exhibit the same size and number of bubbles, whereas bubbles in the LCF composites with the lowest fiber contents (60/40 and 70/30 wt/wt%) were apparently larger than those in the GCF composites with the same PU/fiber ratios.

The CF composites yielded the best results for mechanical properties, which may also be related to smaller bubbles, as shown in the micrographs.

#### 3.4.4. Density

Table 9 shows the calculated (theoretical) and experimentally obtained densities of PU and the composites.

Pure PU showed the highest density (1.1 g/cm^3^) when compared to the composites. This result is consistent with the literature [9], where the density of castor oil-based PU is also reported as 1.1 g/cm^3^.

The lowest density (around 0.8 g/cm^3^) was recorded for PU/LCF composites, corroborating the microscopy results, which indicated a larger number of bubbles in PU/LGF composites.

The density of the PU/CF composites was around 1.0 g/cm^3^. Bubbles in the PU/CF composites were smaller than those in the PU/coir fiber composites (Figure 6, Figure 7 and Figure 8), which likely resulted in higher density in the former.

These findings are in line with the literature [23,29]. Castor oil- and natural fiber-based PU composites exhibit densities between 0.8 and 1.0 g/cm^3^ [23,29]. Castor oil-based PU composites with bamboo particles and 90, 85 and 70 wt/wt% of lignocellulosic filler have densities of 0.9 ± 0.1 g/cm^3^, 1.0 ± 0.0 g/cm^3^ and 0.9 ± 0.1 g/cm^3^, respectively [22].

The theoretical densities of the composites are presented in Table 9. Experimental density was lower than the theoretical values for all the composites, which corroborates the microscopy findings of bubbles in all the composites.

Although theoretically, the densities of PU/LCF and PU/GCF should be the same, differences were observed, likely due to the presence of bubbles identified in microscope analysis.

#### 3.4.5. Water Absorption

The water absorption results are shown in Table 10. Composites with a larger amount of lignocellulosic filler exhibited the highest water absorption. These fibers are hydrophilic, meaning that the lignocellulosic composite absorbs moisture [6]. Marinho et al. [22] studied water absorption in castor oil-based PU and bamboo particle composites with PU contents of 10, 15 and 20 wt/wt%, and reported values of 68.3 ± 12.3%, 33.7 ± 1.9% and 22.9 ± 2.9%, respectively.

This confirms that the higher the PU content of castor oil-based PU, the less water the composite absorbs [1]. Two-component PUs that contain polyols, such as castor oil-based PU, are highly resistant to hydrolysis due to the long hydrophobic chains [9]. The larger the amount of natural fiber, the greater the water absorption [1,6,29,30]. Low water absorption is beneficial in composites used in interior design and civil construction.

Water absorption was higher in the LCF than the LGF composites at all the PU/fiber ratios (50/50, 60/40 and 70/30 wt/wt%).

The lowest water absorption was observed in the PU/GCF composites, particularly those with low fiber contents. This is probably because the drying process may have been more efficient for GCF, since the ground fibers have a larger surface area that facilitates drying. Drying was performed under the same conditions for all the fibers before they were added to the PU.

All the PU/CF composites yielded similar water absorption values for all the compositions analyzed.

#### 3.4.6. Flammability

According to the UL94 standard, used as the basis for flammability testing, burning is only timed from the moment the flame of the Bunsen burner is removed. Since the pure PU test specimen did not continue to burn after the flame was removed, burning could not be timed nor the burn rate calculated for this specimen.

**Figure 9:**
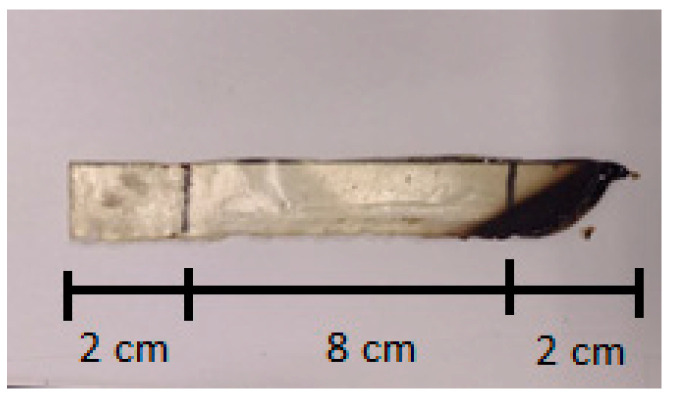
PU test specimen after flammability testing.

Additionally, the flexibility of this specimen means it did not remain horizontal in the sample holder, which also compromised test execution. Figure 9 shows the pure PU test specimen after attempting the test. Table 11 shows the average burning times and burn rates for each test specimen.

For applications in interior design, it is important for composites to show minimal or no inflammability. As such, the longer the average burning time and lower the average burn rate the better, since this indicates that the composite is less flammable.

Average Burning Times

The LCF and GCF composites exhibited almost the same average burning times, except for PU/GCF: 60/40 wt/wt%. The composites with the highest burning times were those containing cellulose fiber, with an increase in fiber content resulting in the longest burning time. In relation to the proportions tested, the composites with longer burning times were those with a PU/fiber ratio of 50/50 wt/wt%, while a ratio of 70/30 wt/wt%, that is, less fiber, produced shorter times. PU decomposes very rapidly, releasing a large amount of heat when exposed to fire [64], and as such, composites with a higher PU percentage were expected to burn faster. The presence of fiber makes the composite less flammable.

Burn Rate

Mathematical analysis of the results indicated very similar burn rates. Burn rates were slightly higher in the LCF composites for all the ratios tested. Additionally, different behaviors were observed for each composition, depending on the type of natural fiber used. In the CF composites, the specimen began twisting as the flame traveled across it, with the burned section forming a spiral.

Table 12 contains photographs taken at burning times of 30, 60 and 90 s of composites with extreme outcomes, namely the lowest (PU/GCF 60/40 wt/wt%) and highest flammability (PU/CF 70/30 wt/wt%).

In the LCF composites, small sparks were observed as the flame progressed along the specimen, with only the burned fiber remaining in this section after burning, while in the GCF composites, the flame caused the formation of a carbonaceous cluster that dripped while still in flames, only going out when it reached the sample holder.

## 4. Subjective Flexibility Analysis of the Composites

A product required to be flexible should also have other satisfactory properties. As such, this section discusses the flexibility of the composites in conjunction with other properties required for applications such as civil construction and interior design. The values obtained (positive, negative or median) in the analysis of the properties of the composites are presented in Table 13.

Positive values for a property are shown in green, negative in red and median in blue. Additionally, in the section that quantifies the number of properties with positive, negative or median values for the different composites, multiplication by 1, 2 or 3 is performed in accordance with the importance of each property predetermined by the methodology.

In general, some mechanical properties generated a high standard deviation but were still used because they were consistent. The burn rates were not used because they did not accurately define the best or worst result.

PU/CF: 60/40 and 70/30 wt/wt% composites are the most recommended for applications that require flexibility, since their properties are more positive (+13 and +12, respectively), with greater emphasis (importance) on flexibility-related properties. The CF composites exhibited better thermal (higher Tonset) and mechanical properties (tensile modulus, toughness and toughness).

Other characteristics of these composites (PU/CF) include their white coloring (Table 3), making it possible to obtain other colors by adding a pigment or dye, and the ability for different prints to be applied on their surface, further expanding their possible uses.

PU/LCF: 60/40 and 70/30 wt/wt% composites also exhibited a positive set of properties (+8 and +7, respectively), with similar coloring to natural wood (Table 4), also appealing to the interior design sector because they resemble a natural renewable material.

GCF composites were the most flexible given their lower flexural modulus and flexural strength values. However, they were also the most fragile, evident in their low toughness and tensile modulus values.

Two composites exhibited median values (+1 and +4) for the set of properties, namely PU/GCF: 70/30 wt/wt% and PU/CF:50/50 wt/wt%, respectively, making them suitable for use in less technically demanding products, such as handcrafts and office items (notebooks, flyleaves and endpapers, pen holders, etc.).

The PU/GCF 50/50 and 40/60 wt/wt% composites exhibited the lowest values for the set of properties studied and are not considered suitable for interior design applications, as was established for PU/LCF: 50/50 wt/wt%.

Both composites (cellulose and coir fiber) have advantages and disadvantages, as already discussed in the literature [65]. In the specific case of the present study, the CF composite, which was more noteworthy from a technical perspective, used cellulose from chemically treated wood [57]. Using chemically treated fiber is an environmental disadvantage when compared to coir fiber, which can be used after fiber separation and drying, a simpler process with no need for chemical treatment [31]. However, cellulose fiber can be obtained from difficult-to-discard household waste, such as disposable diapers. Future research could investigate PU composites containing cellulose fiber from diapers as an alternative approach to add value to this waste product.

## 5. Multicriteria Decision Analysis of the Properties

**Figure 10:**
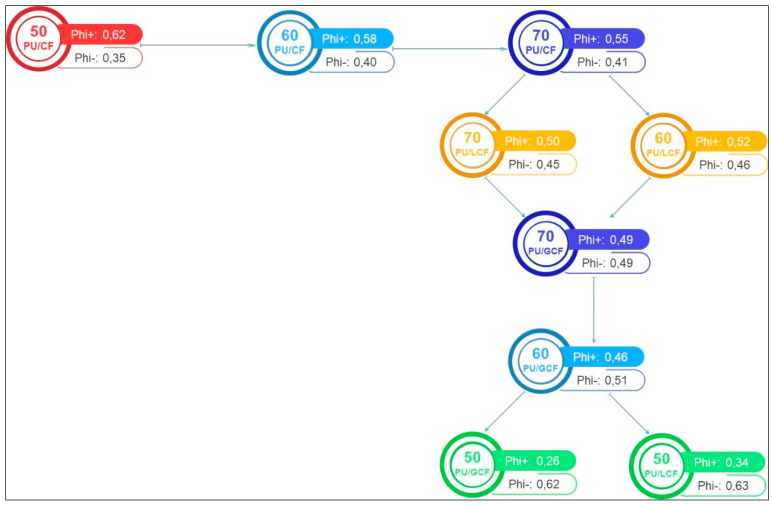
Ranking with the PROMETHEE method.

The PROMETHEE method was used to compare the performance of the properties for each composite by combining their characteristics, with initial results presented in Figure 10.

Figure 10 makes it possible to compare the properties based on the PROMETHEE outranking flow.

✓ Positive outranking flow (*ϕ*+): How one alternative outranks all the others.
✓ Negative outranking flow (*ϕ*−): How one alternative is outranked by all the others.
✓ Net outranking flow (*ϕ*): Difference between the positive and negative flows, used for final classification of the alternatives.

**Figure 11:**
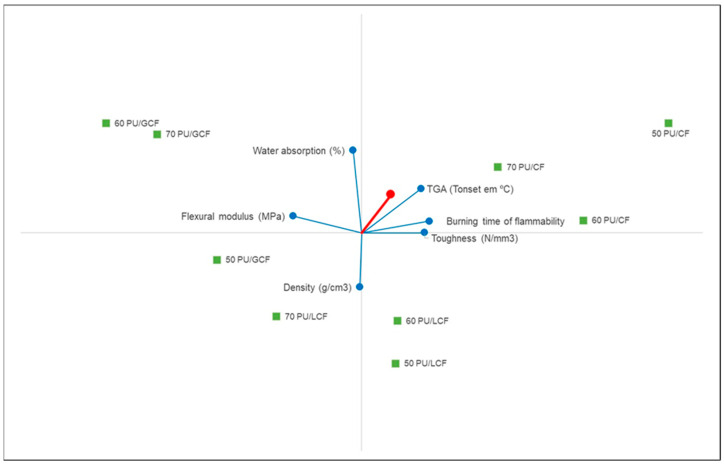
PROMETHEE-GAIA graph.

Figure 11 demonstrates that the PU/CF: 50/50, 60/40 and 70/30 wt/wt% composites showed the greatest value, with net flows of 0.62, 0.58 and 0.55, respectively. This reflects their importance in terms of greater flexibility but also considers the results obtained for the other properties. On the other hand, PU/GCF: 50/50 wt/wt%, with a net flow of 0.26, and PU/LCF: 50/50 wt/wt% at 0.34 were the most overclassified composites. Studying overclassifications is important to identify possibilities for exclusion or inclusion in the decision-making process.

Figure 11 is a geometrical analysis for interactive aid (GAIA) graph of the PROMETHEE method. The GAIA graph is a visual tool used in the multicriteria PROMETHEE method to help analyze and interpret preferences and classifications of the alternatives. It provides a one-dimensional graphical representation of the alternatives and criteria, making it easier to visualize the relationships and trade-offs between them [41].

The PROMETHEE-GAIA graph (Figure 10) visually represents the comparative ranking of the PU/CF, PU/GCF and PU/LCF systems based on multiple criteria. The key conclusions from this graph are as follows:

✓ PU/CF systems (cellulose fiber composites) show the best overall performance:
  ○ The PU/CF 50/50, 60/40 and 70/30 wt/wt% composites achieved the highest net outranking flows, indicating superior flexibility, toughness and thermal stability.
  ○ These composites exhibit greater tensile modulus, toughness and thermal stability values (higher TGA Tonset values).
  ○ Their lower flammability (longer burning time) further reinforces their suitability for flexible applications in interior design.
✓ PU/LCF systems (long coir fiber composites) exhibit moderate performance:
  ○ PU/LCF 60/40 and 70/30 wt/wt% rank just below the PU/CF composites.
  ○ These composites have good mechanical properties (tensile strength, toughness and flexural modulus) but higher water absorption and slightly lower thermal stability than PU/CF.
  ○ Their color and texture resemble natural wood, making them appealing for esthetic applications.
✓ PU/GCF systems (ground coir fiber composites) are more flexible but fragile:
  ○ PU/GCF 50/50 and 60/40 wt/wt% rank the lowest, with net outranking flows of 0.26 and 0.34, respectively.
  ○ These composites display greater flexibility (lower flexural strength and modulus) but also the lowest toughness and tensile modulus values, making them more fragile and less structurally reliable.
  ○ Their low density and high water absorption may limit their use in structural applications but may be suitable for non-load-bearing products such as handcrafted goods.

In short, the GAIA graph is a powerful visual tool that complements the PROMETHEE method and helps decision makers interpret results and understand the dynamics between alternatives (composites) and criteria (properties) clearly and intuitively.

A comparison between the results using both analysis techniques (Table 14) indicated consistency. Only the composites with the highest fiber content (50/50) responded differently to the two techniques. The unevenness of the fibers means that their composites also performed irregularly in the properties assessed, whereby the composites with the highest fiber contents exhibited greater irregularities and a higher likelihood of inconsistency in the assessments.

The preferences, weights and functions used in the PROMETHEE method make analysis more subjective, which may explain the variation in results for the composites.

## 6. Conclusions

The incorporation of natural fibers (coir and cellulose) reduced the flexibility of the plant-based (castor oil) polyurethane matrix. The composites with long fibers and a higher polyurethane percentage showed greater potential for use in products that require flexibility. Adding fiber also increased water absorption and decreased the density of the PU matrix.

The long coir fiber composites showed the best thermal stability, tensile strength, toughness, tensile and flexural moduli, and flexural strength, as well as higher water absorption and density than the ground coir fiber composites.

The ground coir fiber composites were more flexible due to their lower flexural strength and flexural moduli but exhibited the lowest toughness and tensile moduli, making them more fragile.

Cellulose was the best-performing natural fiber to obtain tougher composites with greater tensile strength and thermal stability. Given their coloring, the composites best suited to use in interior design are those containing 40 and 30 wt% of cellulose fiber.

The PROMETHEE method was important to the study because it provided a structured and intuitive approach to decision making. By modeling preferences, attributing weights and enabling pairwise comparison, it provided a detailed personalized analysis of the alternatives (composites). Additionally, the GAIA graph is a visual tool that facilitates interpreting results and communicating decisions, making the process more transparent and understandable. This combination of analytical rigor and visual clarity makes PROMETHEE an important tool to assess and classify alternatives in contexts in which multiple properties must be considered together and equally.

In light of the different results, multicriteria and subjective analysis made it possible to select the composites with the best set of results. And the results of subjective property set analysis were validated by multicriteria analysis, providing a simple analysis for application.

## Figures and Tables

**Table 1:** Size of fibers used in composites.

Fiber	Length (mm)	Width (µm)
Long coir fiber	16.8 ± 3.9	16.7 ± 7.3
Ground coir fiber	0.2 ± 0.0	14.9 ± 3.8
Cellulose fiber	0.8 ± 0.1	3.3 ± 1.4

**Table 2:** Amount by weight of raw material used to prepare the PU and natural fiber composites.

Raw Material	Amount of Raw Material (g)		
	50/50 wt/wt% PU/Fiber	60/40 wt/wt% PU/Fiber	70/30 wt/wt% PU/Fiber
Component A (polyol)	6.68	8.00	9.34
Component B (pre-polymer)	3.34	4.00	4.66
Total amount of PU	10.00	12.00	14.00
Lignocellulosic fiber	10.00	8.00	6.00
Total amount of raw material	20.00	20.00	20.00

**Table 3:** Weights of the properties in PROMETHEE.

Property	Weight
Toughness	4
Flexural strength	4
Flexural modulus	4
TGA (Tonset)	1
Tensile modulus	1
Presence of bubbles by SEM	1
Density	1
Water absorption	1
Burning time in flammability	1

**Table 4:** Holocellulose, α-cellulose, hemicellulose and acid-insoluble lignin content of the coir and cellulose fibers.

Natural Fiber	Holocellulose (%)	α-Cellulose (%)	Hemicellulose (%)	Acid-Insoluble Lignin (%)
Coir fiber	80.5 ± 3.5	52.0 ± 8.5	28.5 ± 8.5	39.0 ± 5.7
Cellulose fiber	93.0 ± 0.1	82.0 ± 5.7	11.0 ± 5.7	2.0 ± 1.4

**Table 5:** Photographs of the plates of PU/fiber composites containing long and ground coir fibers at ratios of 50/50, 60/40 and 70/30 wt/wt%.

Composition (wt/wt%)	PU/Fiber Composite		
	Long Coir Fiber—LCF	Ground Coir Fiber—GCF	Cellulose Fiber—CF
50/50	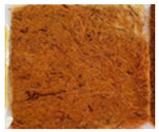	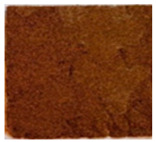	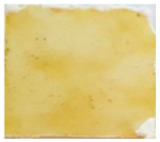
60/40	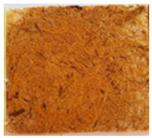	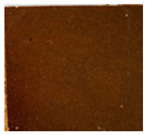	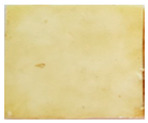
70/30	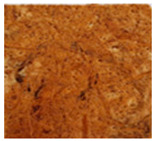	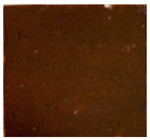	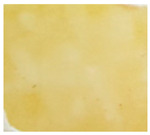

**Table 6:** Initial degradation temperatures (Tonset) and percentage of undegraded waste for the PU/fiber composites.

PU/Fiber Ratio (wt/wt%)	Raw Material	Tonset (°C)	Residue at 800 °C (%)
0/100	LCF	289.2	25.8
	GCF	274.2	24.1
	CF	321.6	9.2
100/0	PU	294.7	2.3
50/50	PU/LCF	294.6	16.1
	PU/GCF	288.8	19.8
	PU/CF	325.6	9.2
60/40	PU/LCF	304.3	12.6
	PU/GCF	292.1	16.8
	PU/CF	317.8	7.8
70/30	PU/LCF	291.4	15.5
	PU/GCF	298.9	15.9
	PU/CF	324.9	8.7

**Table 7:** Toughness and tensile moduli of PU and PU/LCF, PU/GCF and PU/CF composites.

PU/Fiber (wt/wt%)	Raw Material	Toughness (N/mm^3^)	Tensile Modulus (MPa)
50/50	PU/LCF	19.6 ± 14.9	53.1 ± 21.5
	PU/GCF	6.1 ± 2.9	-
	PU/CF	94.7 ± 85.1	248.1 ± 225.4
60/40	PU/LCF	39.5 ± 27.1	76.0 ± 47.5
	PU/GCF	9.9 ± 2.5	9.5 ± 2.1
	PU/CF	84.7 ± 41.1	220.9 ± 83.3
70/30	PU/LCF	62.7 ± 58.5	211.3 ± 174.2
	PU/GCF	5.3 ± 4.8	11.5 ± 2.2
	PU/CF	49.9 ± 44.2	80.5 ± 46.7
PU		200.9 ± 61.1	3.7 ± 0.2

**Table 8:** Flexural strength of PU and PU/LCF, PU/GCF and PU/CF composites.

PU/Fiber (wt/wt%)	Raw Material	Flexural Strength (MPa)	Flexural Modulus (MPa)
50/50	PU/LCF	16.4 ± 7.9	243.6 ± 108.9
	PU/GCF	4.6 ± 3.2	81.2 ± 47.8
	PU/CF	25.2 ± 5.4	330.9 ± 272.6
60/40	PU/LCF	11.4 ± 6.1	86.3 ± 91.8
	PU/GCF	2.2 ± 0.7	37.8 ± 12.9
	PU/CF	18.2 ± 3.5	248.5 ± 49.5
70/30	PU/LCF	10.0 ± 5.3	81.2 ± 45.0
	PU/GCF	4.4 ± 0.5	71.7 ± 37.2
	PU/CF	11.6 ± 1.8	163.0 ± 42.1

**Table 9:** Experimental and estimated theoretical densities of PU and PU/LCF, PU/GCF and PU/CF composites.

PU/Fiber (wt/wt%)	Raw Material	Density (g/cm^3^)	Theoretical Density (g/cm^3^)
50/50	PU/LCF	0.8 ± 0.1	1.2
	PU/GCF	1.0 ± 0.0	1.2
	PU/CF	1.0 ± 0.1	1.3
60/40	PU/LCF	0.8 ± 0.1	1.2
	PU/GCF	1.0 ± 0.0	1.2
	PU/CF	1.0 ± 0.0	1.3
70/30	PU/LCF	0.8 ± 0.0	1.2
	PU/GCF	0.9 ± 0.1	1.2
	PU/CF	1.0 ± 0.0	1.2
	PU	1.1 ± 0.0	

**Table 10:** Water absorption of PU and PU/LCF, PU/GCF and PU/CF composites.

PU/Fiber (wt/wt%)	Raw Material	Water Absorption (%)
50/50	PU/LCF	45.4 ± 0.5
	PU/GCF	30.0 ± 0.2
	PU/CF	17.4 ± 1.1
60/40	PU/LCF	37.7 ± 0.1
	PU/GCF	7.5 ± 0.1
	PU/CF	20.3 ± 2.9
70/30	PU/LCF	21.5 ± 4.9
	PU/GCF	6.3 ± 0.7
	PU/CF	21.1 ± 1.5
	PU	0.7 ± 0.1

**Table 11:** Average burning times and burn rates in flammability testing for PU/natural fiber composites.

PU/Fiber (wt/wt%)	Raw Material	Average Buring Time (s)	Average Burn Rate (mm/s)
50/50	PU/LCF	111.8 ± 21.4	53.7 ± 11.1
	PU/GCF	98.3 ± 42.2	32.4 ± 6.8
	PU/CF	197.3 ± 21.1	30.4 ± 3.2
60/40	PU/LCF	123.6 ± 9.2	42.7 ± 13.9
	PU/GCF	76.8 ± 47.9	25.8 ± 8.7
	PU/CF	165.9 ± 20.4	36.2 ± 4.3
70/30	PU/LCF	90.8 ± 17.5	75.9 ± 66.1
	PU/GCF	91.9 ± 42.4	41.1 ± 13.6
	PU/CF	146.7 ± 53.7	40.9 ± 8.1

**Table 12:** Photographs of the PU/GCF (60/40 wt/wt%) and PU/CF composites (70/30 wt/wt%) at 30, 60 and 90 s of the flammability test.

PU/Fiber (wt/wt%)	Burning Time		
	t = 30 s	t = 60 s	t = 90 s
PU/GCF (60/40)	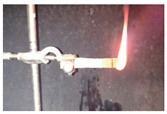	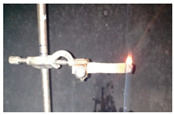	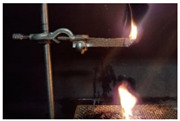
PU/CF (70/30)	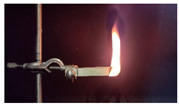	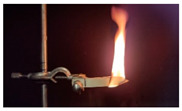	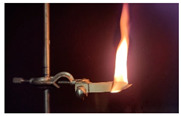

Legend: PU = polyurethane; CF = cellulose fiber; GCF = ground coir fiber.

**Table 13:** Summary of the main properties of the composites and assessment of the composition that performed best for the set of properties.

Property	PU/LCF (wt/wt%)			PU/GCF (wt/wt%)			PU/CF(wt/wt%)			Mean
	50/50	60/40	70/30	50/50	60/40	70/30	50/50	60/40	70/30	
Toughness (N/mm^3^)	20	40	63	6	10	5	95	85	50	41
Flexural strength (MPa)	16	11	10	5	3	4	25	18	12	12
Flexural modulus (MPa)	244	86	82	82	38	72	331	249	163	149
TGA (Tonset in °C)	295	304	292	289	292	299	326	318	325	304
Tensile modulus (MPa)	53	76	211	−	10	12	248	221	81	101
Presence of bubbles in SEM	−1	−1	−1	−1	−1	−1	+1	+1	+1	
Density (g/cm^3^)	0.8	0.8	0.8	1.0	1.0	0.9	1.0	0.9	1.0	0.9
Water absorption (%)	45	38	22	30	8	6.3	17	20	21	23
Burning time in flammability (s)	112	124	91	98	77	92	197	166	147	123
Number of properties (p.) with more positive, negative or median values according to the mean for each property										
Number of positive properties for flexibility (×3)	1p. = +3	1p. = +3	2p. = + 6	2p. = +6	2p. = +6	2p. = +6	1p. = +3	2p. = +6	0p. = 0	
Number of negative properties for flexibility (×3)	2p. = −6	0p. = 0	0p. = 0	1p. = −3	1p. = −3	1p. = −3	2p. = −6	1p. = −3	0p. = 0	
Number of properties with median value for flexibility (×2)	0p. = 0	2p. = + 4	1p. = +2	0p. = 0	0p. = 0	0p. = 0	0p. = 0	0p. = 0	3p. = +6	
Number of other positive properties (×2)	1p. = +2	1p. = + 2	2p. = +4	0p. = 0	1p. = +2	1p. = +2	4p. = +8	4p. = +8	3p. = +6	
Number of other negative properties (×2)	3p. =−6	2p. = −4	3p. = −6	4p. = −8	5p. = −10	3p. = −6	1p. = −2	0p. = 0	1p. = −2	
Number of other properties with median value (×1)	2p. = + 2	3p. = + 3	1p. = +1	1p. = +1	0p. = +0	2p. = +2	1p. = +1	2p. = +2	2p. = +2	
Sum of the values	−5	+8	+7	−4	−5	+1	+4	+13	+12	

Green: most positive value for the property; red: most negative value for the property; blue: median value for the property in relation to the mean; properties related to flexibility are in gray; in the presence of bubbles, more positive (+1) and more negative (−1) values were used for MCDA calculations.

**Table 14:** Composite classification by subjective and multicriteria analysis based on a set of more positive properties.

Classification in descending order of the set of properties in subjective analysis	More positive results 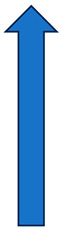 More negative results	Classification in descending order of the set of properties in multicriteria analysis
PU/CF: 60/40		5. PU/CF: 50/50
2. PU/CF: 70/30		1. PU/CF: 60/40
3. PU/LCF: 60/40		2. PU/CF: 70/30
4. PU/LCF: 70/30		3. PU/LCF: 60/40
5. PU/CF: 50/50		4. PU/LCF: 70/30
6. PU/GCF: 70/30		6. PU/GCF: 70/30
7. PU/GCF: 50/50		8. PU/GCF: 60/40
8. PU/GCF: 60/40		9. PU/LCF: 50/50
9. PU/LCF: 50/50		7. PU/GCF: 50/50

## Data Availability

The original contributions presented in this study are included in the article. Further inquiries can be directed to the corresponding author.

## References

[1] Sanjay M.R., Madhu P., Jawaid M., Senthamaraikannan P., Senthil S., Pradeep S. (2018). Characterization and properties of natural fiber polymer composites: A comprehensive review. J. Clean. Prod..

[2] Karthi N., Kumaresan K., Sathish S., Gokulkumar S., Prabhu L., Vigneshkumar N. (2020). An overview: Natural fiber reinforced hybrid composites, chemical treatments and application areas. Mater. Today Proc..

[3] Karimah A., Ridho M.R., Munawar S.S., Adi D.S., Ismadi, Damayanti R., Subiyanto B., Fatriasari W., Fudholi A. (2021). A review on natural fibers for development of eco-friendly bio-composite: Characteristics, and utilizations. J. Mater. Res. Technol..

[4] Elfaleh I., Abbassi F., Habibi M., Ahmad F., Guedri M., Nasri M., Garnier C. (2023). A comprehensive review of natural fibers and their composites: An eco-friendly alternative to conventional materials. Results Eng..

[5] Väisänen T., Haapala A., Lappalainen R., Tomppo L. (2016). Utilization of agricultural and forest industry waste and residues in natural fiber-polymer composites: A review. Waste Manag..

[6] Väisänen T., Das O., Tomppo L. (2017). A review on new bio-based constituents for natural fiber-polymer composites. J. Clean. Prod..

[7] Karimah A., Ridho M.R., Munawar S.S., Ismadi, Amin Y., Damayanti R., Lubis M.A.R., Wulandari A.P., Nurindah I.A.H., Fudholi A. (2021). A Comprehensive Review on Natural Fibers: Technological and Socio-Economical Aspects. Polymers.

[8] Cicconi P. (2020). Eco-design and Eco-materials: An interactive and collaborative approach. Sustain. Mater. Technol..

[9] Kaur R., Singh P., Tanwar S., Varshney G., Yadav S. (2022). Assessment of bio-based polyurethanes: Perspective on applications and biodegradation. Macromol.

[10] Atigah A., Mastura B.A., Ali A., Jawasai M., Sapuan S.M. (2017). A review on Polyurethane and its Polymer Composites. Curr. Org. Synth..

[11] Lopes M.D.M., Padua M.S., Carvalho J.P.R.G., Simonassi N.T., Lopez F.P.D., Colorado H.A., Vieira C.M.F. (2021). Natural based polyurethane matrix composites reinforced with bamboo fiber waste for use as oriented strand board. J. Mater. Res. Technol..

[12] Temer B.C., D’Almeida J.R.M. (2015). Development and Characterization of Agglomerated Panels Using Residues from the Sustainable Production of Heart of Palm from Pejibaye (*Bactris gasipaes*) Palms. Polym. Renew. Resour..

[13] Bhaskaran S.K., Boga K., Arukula R., Gaddam S.K. (2023). Natural fibre reinforced vegetable-oil based polyurethane composites: A review. J. Polym. Res..

[14] Pinto E.R.P., Barud H.S., Silva R.R., Palmieri M., Polito W.L., Calil V.L., Cremona M., Ribeiro S.J.L., Messaddeq Y. (2015). Transparent composites prepared from bacterial cellulose and castor oil based polyurethane as substrates for flexible OLEDs. J. Mater. Chem. C.

[15] Zhao Z., Li B., Ma P. (2023). Advances in mechanical properties of flexible textile composites. Compos. Struct..

[16] Canevarolo S.V. (2002). Ciência dos Polímeros. Um Texto Básico Para Tecnólogos e engenheiros (In English: Polymer Science. A Basic Text for Technologists and Engineers).

[17] Liu W., Xie T., Qiu R. (2015). Styrene-free unsaturated for hemp fiber composite. Compos. Sci. Technol..

[18] Liu W., Xie T., Qiu R. (2016). Biobased thermosets prepared from rigid isosorbide and flexible soybean oil derivates. ACS Sustain. Chem. Eng..

[19] Hammed N., Salim N.V., Walsh T.R., Wiggins J.S., Ajayan P.M., Fox B.L. (2015). Ductile thermoset polymers by controlling network flexibility. Chem. Comm..

[20] Merlini C., Soldi V., Barra G.M.O. (2011). Influence of fiber surface treatment and length on physio-chemical properties of short random banana fiber-reinforced castor oil polyurethane composite. Polym. Test..

[21] Liu W., Chen T., Fei M., Qiu R., Yu D., Fu T., Qiu J. (2019). Properties of natural fiber-reinforced bio based thermoset biocomposites: Effects of fiber type and resin composition. Compos. B Eng..

[22] Marinho N.P., Nascimento E.M., Nisgoski S., Magalhães W.L.E., Neto S.C., Azevedo E.C. (2013). Physical and thermal characterization of polyurethane based on castor oil composite with bamboo particles. Polímeros.

[23] Sánchez M.L., Morales L.Y., Caicedo J.D. (2017). Physical and mechanical properties of agglomerated panels made from bamboo fiber and vegetable resin. Constr. Build. Mater..

[24] Sánchez M.L., Capote G., Carrillo J. (2019). Composites reinforced with Guadua fibers: Physical and mechanical Properties. Constr. Build. Mater..

[25] Mothé C.G., Araujo C.R. (2004). Thermal and mechanical caracterization of polyuretane composites with Curaua fibers. Polímeros.

[26] Mothé C.G., Araujo C.R., Wang S.H. (2009). Thermal and mechanical characteristics of polyurethane/curaua fiber composites. J. Therm. Anal. Calorim..

[27] Guimarães J.L., Frollini E., Silva C.G., Wypych F., Satyanarayanac K.G. (2009). Characterization of banana, sugarcane bagasse and sponge gourd fibers of Brazil. Ind. Crops Prod..

[28] Chen Y.-H., Wu C.-H., Chen Y.-C. (2021). Optimized condition for eco-friendly wood composites manufactured from castor oil-based polyurethane. Constr. Build. Mater..

[29] Oliveira J.N., Lopes F.P.D., Simonassi N.T., Souza D., Monteiro S.N., Vieira C.M.F. (2023). Evaluation of the physical properties of composite panels with eucalyptus sawdust waste and castor oil-based polyurethane. J. Mater. Res. Technol..

[30] Faria D.L., Mesquita L., Resende A.A., Lopes D.E., Mendes L.M., Martins M.A., Marconcini J.M., Guimarães J.B. (2020). Physical and mechanical properties of polyurethane thermoset matrices reinforced with green coconut fibers. J. Compos. Mater..

[31] Hasan K.M.F., Horváth P.G., Bak M., Alpár T. (2021). A state-of-the-art review on coir fiber-reinforced biocomposites. RSC Adv..

[32] Glowinska E., Datta J., Parcheta P. (2017). Effect of sisal fiber filler on thermal properties of bio-based polyurethane composites. J. Therm. Anal. Calorim..

[33] AL-Oqla F.M., Sapuan S.M., Ishak M.R. (2016). Nuraini AA. A decision-making model for selecting the most appropriate natural fiber—Polypropylene-based composites for automotive applications. J. Compos. Mater..

[34] Al-Shamary K.J., Yas Q.M., Badr A.M., Shalabi R.A., Aldulaimi S.H. Multi Criteria Decision Making Technique for Evaluation and Selection performance Large Scale Data of Composite Materials. Proceedings of the ASU International Conference in Emerging Technologies for Sustainability and Intelligent Systems (ICETSIS).

[35] Kumar D., Marchi M., Alam S.B., Kavka C., Koutsawa Y., Rauchs G., Belouettar S. (2022). Multi-criteria decision making under uncertainties in composite materials selection and design. Compos. Struct..

[36] Noryani M., Sapuan S., Mastura M.T. (2018). Multi-criteria decision-making tools for material selection of natural fibre composites: A review. J. Mech. Eng. Sci..

[37] Milani A., Eskicioglu C., Robles K., Bujun K., Hosseini-Nasab H. (2010). Multiple criteria decision making with life cycle assessment for material selection of composites. Express Polym. Lett..

[38] Alaaeddin M.H., Sapuan S.M., Zuhri M.Y.M., Zainudin E.S., AL-Oqla F.M. (2019). Polymer matrix materials selection for short sugar palm composites using integrated multi criteria evaluation method. Compos. B Eng..

[39] Singh T., Fekete I., Jakab S., Lendvai L. (2023). Selection of straw waste reinforced sustainable polymer composite using a multi-criteria decision-making approach. Biomass Convers. Biorefin..

[40] Kelleci O., Aydemir D., Altuntas E., Kurt R., Oztel A., Yorur H., Istek A. (2022). Wood Flour-Reinforced Green Composites: Parameter Optimization via Multi-criteria Decision-Making Methods. J. Polym. Environ..

[41] Brans J.-P., Mareschal B., Figueira J., Greco S., Ehrgott M. (2005). PROMETHEE Methods. Multiple Criteria Decision Analysis: State of the Art Surveys.

[42] Roy B. (1996). Multicriteria Methodology for Decision Aiding.

[43] Dias L., Mousseau V. (2003). IRIS—Interactive Robustness Analysis and Parameter’s Inference for Multiple Criteria Sorting Problems (Version 2.0)—User Manual. Document of INESC Coimbra, 1. https://hal.science/hal-00004145.

[44] Imperveg Technical Sheet of Resin from Vegetal IMPERVEG RM 122®. www.imperveg.com.br.

[45] Escócio V.A., Pacheco E.B.A.V.P., Souza A.M.F., Brígida M.A.C.S., Soares A.G., Visconte L.L.Y. (2017). Study of natural fibers from waste from sponge gourd, peach palm tree and papaya pseudstem. Int. J. Environ. Agric. Res..

[46] Acha B.A., Carlsson L.A. (2013). Evaluation of cure state of vinylester resins. J. Appl. Polym. Sci..

[47] Oh G. (2022). A simplified toughness estimation method based on standard tensile data. Int. J. Press. Vessels Pip..

[48] Hasan K.M., Horváth P.G., Kóczán Z., Alpár T. (2021). Thermo-mechanical properties of pretreat coir fiber and fibrous chips reinforced multilayered composites. Sci. Rep..

[49] Marinelli A.L., Monteiro M.R., Ambrósio J.D., Branciforti M.C., Kobayashi M., Nobre A.D. (2008). Development of Polymeric Composites with Natural Fibers: A Contribution to the Sustainability of Amazon. Polimeros.

[50] Duinker P.N., Greig L.A. (2007). Scenario analysis in environmental impact assessment: Improving explorations of the future. Environ. Impact Assess. Rev..

[51] Morris P., Therivel P. (2001). Methods of Environmental Impact Assessments.

[52] Tukker A. (2000). Life cycle assessment as a tool in environmental impact assessments. Environ. Impact Assess. Rev..

[53] Keller H.R.M., Massart D.L., Brans J.-P. (1991). Multicriteria decision making: A case study. Chemom. Intell. Lab. Syst..

[54] Bhatia S.K., Smith J.L. (2008). Bridging the Gap Between Engineering and the Global Word—A case study of the coconut (coir) fiber industry in Kerala, India. Synth. Lec. Eng. Technol. Soc..

[55] Corradini E., Rosa M.F., Macedo B.P., Paladin P.D., Mattoso L.H.C. (2009). Chemical composition, thermal and mechanical properties for cultivars of immature coconut fibers. Rev. Bras. Frutic..

[56] Ishizaki M.H., Visconte L.L.Y., Furtado C.R.G., Leite M.C.A.M., Leblanc J.L. (2006). Mechanical and morphological characterization of polypropylene and green coconut fiber composites: Influence of fiber content and mixture conditions. Polimeros.

[57] Chopra L., Manikanika (2022). Extraction of cellulosic fibers from the natural resources: A short review. Mater. Today Proc..

[58] Zhang H., Fu S., Chen Y. (2020). Basic understanding of the color distinction of lignin and the proper selection of lignin in color-depended utilizations. Int. J. Biol. Macromol..

[59] Moshi A.A.M., Ravindran D., Bharathi S.R.S., Padma S.R., Indran S., Divya D. (2020). Characterization of natural cellulosic fiber extracted from Grewia damine flowering plant’s stem. Int. J. Biol. Macromol..

[60] Balasubramanian B., Raja K., Kumar V.V., Ganeshan P. (2024). Characterization study of Holoptelea Integrifolia tree bark fibers reinforced epoxy composites. Nat. Prod. Res..

[61] Lazzari L.K., Neves R.M., Vanzetto A.B., Zattera A.J., Santana R.M.C. (2022). Thermal degradation kinetics and lifetime prediction of cellulose biomass cryogels reinforced by its pyrolysis waste. Mater. Res..

[62] Hadjadj A., Jbara O., Tara A., Gilliot M., Malek F., Maafi E.M., Tighzert L. (2016). Effects of cellulose fiber content on physical properties of polyurethane based composites. Compos. Struct..

[63] Singh M., Singh P., Brar G.S. (2019). Review on natural fiber reinforced Polyurethane composite. Int. J. Emerg. Technol. Innov. Res..

[64] Visakh P.M., Semkin A.O., Rezaev I.A., Fateev A.V. (2019). Review on soft polyurethane flame retardant. Constr. Build. Mater..

[65] Thakur V.K., Thakur M.K. (2014). Processing and characterization of natural cellulose fibers/thermoset polymer composites. Carbohydr. Polym..

